# Sex-specific associations of the plasma proteome with incident coronary artery disease

**DOI:** 10.1093/cvr/cvag186

**Published:** 2026-09-03

**Authors:** Vincent Q Sier, Kristina Dimitrova, Erna H A B Peters, Wouter P M van der Loo, Ko Willems van Dijk, Diana van Heemst, Wilhelmina H Bax, Paul H A Quax, Gerard Pasterkamp, Michal Mokry, J Wouter Jukema, Raymond Noordam, Margreet R de Vries

**Affiliations:** Department of Surgery, Leiden University Medical Center, Albinusdreef 2, 2333ZA Leiden, The Netherlands; Einthoven Laboratory for Experimental Vascular Medicine, Leiden University Medical Center, Leiden, The Netherlands; Central Diagnostic Laboratory, University Medical Center Utrecht, Utrecht University, Heidelberglaan 100, 3508, GA, Utrecht, The Netherlands; Department of Surgery, Leiden University Medical Center, Albinusdreef 2, 2333ZA Leiden, The Netherlands; Einthoven Laboratory for Experimental Vascular Medicine, Leiden University Medical Center, Leiden, The Netherlands; Department of Cardiology, Leiden University Medical Center, Leiden, The Netherlands; Einthoven Laboratory for Experimental Vascular Medicine, Leiden University Medical Center, Leiden, The Netherlands; Department of Human Genetics, Leiden University Medical Center, Leiden, The Netherlands; Department of Internal Medicine, Division of Endocrinology, Leiden University Medical Center, Leiden, The Netherlands; Department of Internal Medicine, Section of Gerontology and Geriatrics, Leiden University Medical Center, Leiden, The Netherlands; Department of Cardiology, Leiden University Medical Center, Leiden, The Netherlands; Department of Surgery, Leiden University Medical Center, Albinusdreef 2, 2333ZA Leiden, The Netherlands; Einthoven Laboratory for Experimental Vascular Medicine, Leiden University Medical Center, Leiden, The Netherlands; Central Diagnostic Laboratory, University Medical Center Utrecht, Utrecht University, Heidelberglaan 100, 3508, GA, Utrecht, The Netherlands; Central Diagnostic Laboratory, University Medical Center Utrecht, Utrecht University, Heidelberglaan 100, 3508, GA, Utrecht, The Netherlands; Laboratory of Experimental Cardiology, University Medical Center Utrecht, Utrecht University, Heidelberglaan 100, 3508, GA, Utrecht, The Netherlands; Department of Cardiology, Leiden University Medical Center, Leiden, The Netherlands; Netherlands Heart Institute, Utrecht, The Netherlands; Health Campus the Hague/Public Health and Primary Care, Leiden University Medical Center, The Hague/Leiden, The Netherlands; Department of Clinical Epidemiology, Leiden University Medical Center, Leiden, The Netherlands; Department of Surgery, Leiden University Medical Center, Albinusdreef 2, 2333ZA Leiden, The Netherlands; Einthoven Laboratory for Experimental Vascular Medicine, Leiden University Medical Center, Leiden, The Netherlands; Department of Surgery, Brigham & Women's Hospital and Harvard Medical School, 75 Francis Street, Boston, MA 02115, USA

**Keywords:** Sex differences, Proteomics, Coronary artery disease, Atherosclerosis, Mendelian randomization

## Abstract

**Aims:**

The aetiology of coronary artery disease (CAD) appears different for men and women, yet insights into underlying sex-specific biological mechanisms are limited. We integrated genomic and proteomic analyses to investigate sex-specific associations of the plasma proteome with CAD.

**Methods and results:**

In 40 829 UK Biobank participants (free-of-CAD, baseline 365 days thereafter; 55% women; mean age 56.9 ± 8.1 years), we examined associations between 2922 plasma proteins and incident CAD over a median follow-up of 13.7 years (IQR 13.1–14.4) using multivariable-adjusted Cox proportional hazards models. Sex-specific analyses identified 440 female exclusive and 32 male exclusive proteins associated with incident CAD (FDR-corrected *P* < 0.05), revealing distinct pathway enrichments, including innate immune response in women and angiogenesis in men. Causality was assessed through combined and sex-stratified two-sample Mendelian randomization (MR) using inverse-variance-weighted analyses with genome-wide association summary statistics from 422 108 men (61 969 cases) and 521 695 women (27 128 cases) (UK Biobank, FinnGen freeze 9). Integration of direct sex-protein interaction analyses with sex-combined MR identified 59 proteins with evidence for sex-specific causal effects. Four proteins demonstrated concordant directionality in sex-stratified MR analyses (*n* = 943 803) and multivariable regression models, namely CDKN2D, MYH9, and SKAP2 (women) and CTSH (men). To assess translational relevance, prioritized targets were further evaluated in secondary major adverse cardiovascular events among carotid endarterectomy patients (MACE; Athero-Express) and acute myocardial infarction (AMI; MISSION!) using plasma proteomics and ELISA. After further top-target identification in the context of MACE and AMI, clinical drug candidates were identified through a machine learning framework, including CTSH (men) and TNFRSF4 (both sexes).

**Conclusion:**

We identified sex-specific associations of proteins and biological pathways with incident CAD. Whereas the majority of proteins had consistent associations in both men and women, our findings suggest a degree of sex-specific pathogenesis with evidence for potential causality, opening new alleys for tailored prevention strategies and clinical cardiovascular risk management.

## Graphical Abstract

**Graphical Abstract:**
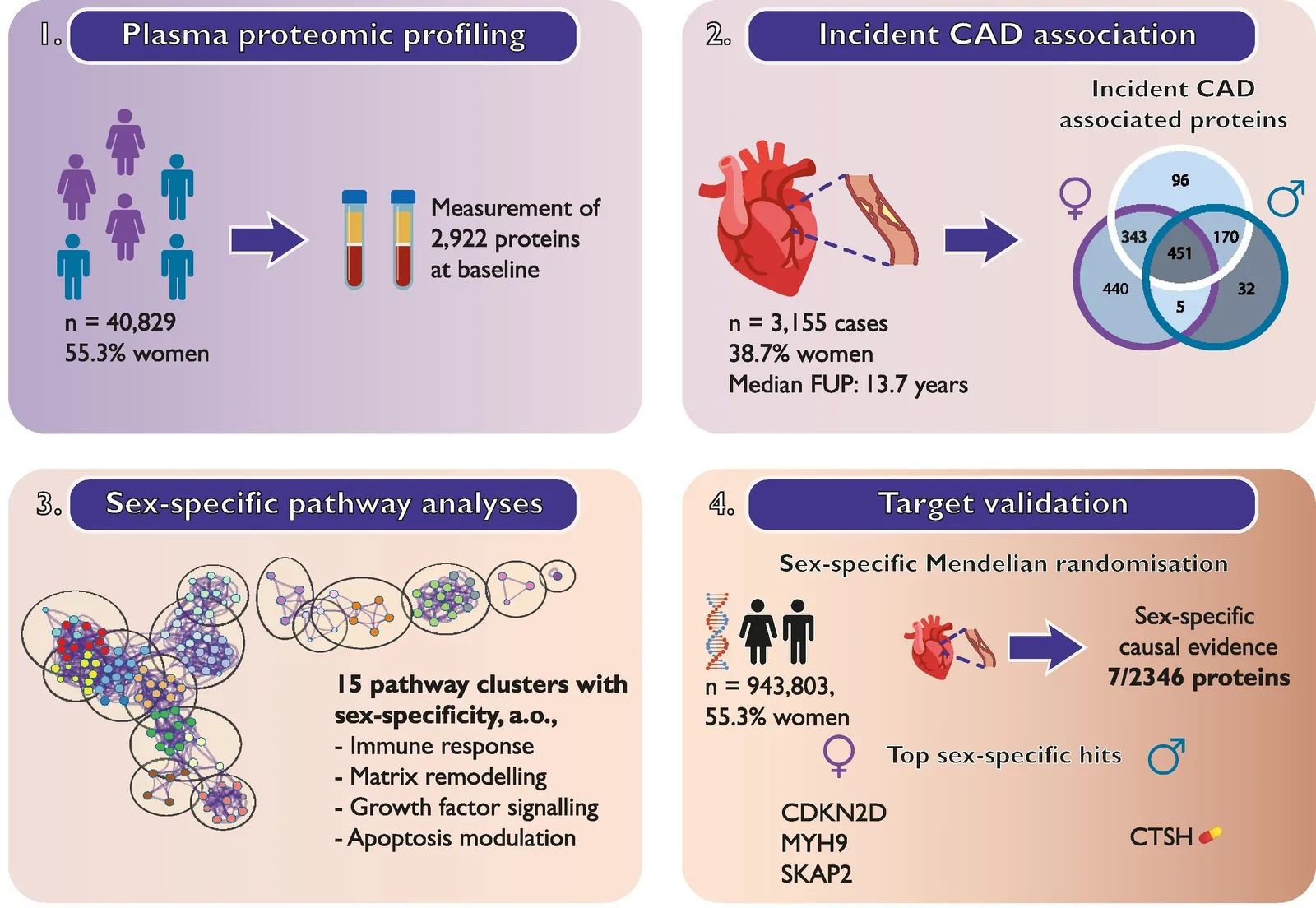

**Time of primary review: 74 days**

## 1. Introduction

Despite the growing recognition of disparities between men and women in the development, manifestation and epidemiology of coronary artery disease (CAD),^1–3^ a critical gap persists in our understanding of its underlying sex-specific biological mechanisms. Both men and women are susceptible to develop ischaemic heart disease upon exposure to traditional risk factors. However, sex-specific aetiologies have been observed, with factors such as smoking, diabetes and stress disproportionally affecting CAD incidence in women.^4,5^ Clinically, women present significantly more frequently with myocardial ischaemia with nonobstructive coronary arteries (INOCA), whereas men present more commonly with classical obstructive CAD.^6^ Regarding the latter, differences are observed in plaque composition, vulnerability and burden between men and women, pointing towards a more atheromatous vs. fibrous phenotype.^7,8^

The plasma proteome reflects the dynamic biological state, which, prior to disease, may provide insight into the mechanisms of (sex-specific) CAD onset. The UK Biobank is a large biomedical healthcare database comprising population-scale data of 2922 unique predetermined plasma proteins in 54 219 individuals.^9^ Illustratively, using the UK Biobank data, a recent study demonstrated the significance of large-scale integration of plasma protein levels and genomics.^10^

Omics approaches, including genomics, transcriptomics, metabolomics and proteomics, are powerful tools to unravel underlying biological mechanisms of disease and may also provide insights into the sex-specific onset of CAD.^11,12^ A limited number of genomic studies have identified multiple sex-specific candidate genes as risk factors for CAD, within risk loci that encode for proteins that were primarily engaged in processes involved in lipid metabolism and vascular remodelling.^13–15^ Recently, the genetic architecture for protein levels appeared similar between men and women,^16^ however, it is still possible that the impact of higher or lower protein levels results in different future atherosclerotic disease risks. Focusing on atherosclerotic lesion development, human regulatory network analyses using patient material have indicated the existence of sex-specific plaque sub-phenotypes and cell signatures.^7,17,18^

Translating experimental findings into the identification of druggable targets is vital to enable clinical application. With the advent of increasingly complex datasets, advanced computational tools like interaction network analyses and artificial intelligence approaches facilitate the translation of novel targets to the clinic.^19^

We hypothesized that distinct proteomic patterns and pathways are associated with CAD onset in men and women. Therefore, in this study, by integrating multivariable-adjusted regressions in combination with causal effect-estimating Mendelian Randomization (MR) approaches,^20^ we aimed to investigate sex-specific plasma proteomic associations with incident CAD and identify potentially causal and druggable protein targets.

## 2. Methods

### 2.1 Statistical analysis

An overview of all included study data and settings is available in Supplementary material online, *Table S1*.

#### 2.1.1 Multivariable-adjusted regression

##### 2.1.1.1. Study setting

The research aim was addressed in UK Biobank, a general population cohort in which approximately 500 000 participants were prospectively followed after recruitment between 2006 and 2010 (https://www.ukbiobank.ac.uk/).^9^ Participants were between 40 and 70 years of age at enrolment and recruited from the general population. Recruitment took place via invitation letters to eligible adults registered to the National Health Services (NHS) and living within 25 miles from one of the assessment centres. Written informed consent was retrieved from all participants, and ethical approval of the study was given by the North-West MultiCentre Ethics Committee. The present study was accepted and completed under project 56340.

##### 2.1.1.2. Study population

The UK Biobank Pharma Plasma Proteome (UKB-PPP) project is a collaboration between thirteen biopharmaceutical companies and the UK Biobank, with the purpose of studying blood protein biomarkers in relation to disease onset. Baseline plasma samples from 54 219 individuals were selected randomly (*n* = 46 595) or by consortium member pre-selection (*n* = 6376) and COVID-19 repeat imaging study (*n* = 1268) protocols. A total of 20 participants were pre-selected by the consortium members and participated in the COVID-19 repeat study. For the current study, we restricted the analysis to participants of the UK Biobank of European ancestry to minimize population stratification bias. Moreover, individuals with a history of CAD or CAD incidence within 365 days after baseline were excluded for all multivariable-adjusted regression analyses to minimize potential influences caused by reverse causation, resulting in a total cohort of 40 829 participants.

##### 2.1.1.3. Exposure

Proteomic profiling on the UKB-PPP samples was performed using the Olink Explore 3072, which processes 2941 protein analytes, corresponding to 2923 unique proteins from the Cardiometabolic, Cardiometabolic_II, Inflammation, Inflammation_II, Neurology, Neurology_II, Oncology and Oncology_II panels. Further details on the Olink Explore platform and assays are available in the summary publication of the UKB-PPP.^10^ Recently, studies have shown that the genetic architecture of the protein levels was similar between men and women, supporting the use of the sex-combined study that has more statistical power.^16^ The current analyses have been performed in accordance with the quality consideration as described in Sun et al., excluding one protein (GLIPR1), due to >80% of data failing quality control (99.4%).^10^

Validation of this platform has been performed previously on random plasma samples of HERITAGE and the Jackson Heart Study, showing moderate correlation (ρ = 0.64–0.87) between the Olink affinity platform and validated commercial ELISAs.^21^

##### 2.1.1.4. Cardiovascular disease outcomes

A UK Biobank algorithm was employed to collect participant data on incident CAD, using data from the general practitioner, linked hospital admissions, death registries and self-report. As coded according to the International Classification of Diseases (ICD) standards, the study outcome of CAD was defined as angina pectoris (I20), myocardial infarction (I21, I22) and acute and chronic ischaemic heart disease (I24, I25). Participants were followed until the occurrence of the first CAD event, death, loss to follow-up or the end of follow-up, whichever came first.

##### 2.1.1.5. Other variables

Data on age, sex, body mass index (BMI), insulin use, smoking, menopausal status (for women only) and use of cholesterol-lowering medication were collected at the assessment centre of the UK Biobank through a touchscreen questionnaire and physical measures. For smoking status, we included previous and current smokers. The Townsend Deprivation Index (TDI) was included as a marker for socio-economic status and reflects the neighbourhood of the individual participant based on zip code. Non-insulin-dependent type diabetes mellitus was obtained via linkage to health-related records.

##### 2.1.1.6. Multivariable-adjusted Cox-proportional hazard models

All the statistics were performed in R version 3.6.1 statistical software (the R Foundation for Statistical Computing, Vienna, Austria). Characteristics of the study population were studied at baseline as means (with standard deviations), medians (with inter-quartile ranges; for non-normally distributed continuous variables), or proportions (for categorical variables only).

Cox-proportional hazard models were performed to investigate the associations between standardized plasma protein levels (in standard deviation increase in level) and incident CAD (Survival package in R^22^). In these analyses, participants were observed until the end of the follow-up period, loss to follow-up or disease development, whichever came first. These analyses were adjusted for baseline age, sex, BMI, TDI, baseline diagnosis of diabetes mellitus, insulin use, smoking and use of cholesterol-lowering medication. Blood samples in our cohort were collected at baseline and therefore reflect plasma lipid profiles at years prior to incident CAD events. Therefore, instead of adjusting for a single (randomly collected) assessment of plasma lipids and blood pressure, we considered elevated cardiovascular risk as baseline use of anti-hypertensive and cholesterol-lowering medication use. Analyses were performed for the total study population, as well as stratified for men and women. Additional female-restricted analyses were performed to adjust and additionally restrict for post-menopausal status. Moreover, additional analyses were performed to consider blood cell counts (UK Biobank: 30160, 30150, 30120, 30130, 30140, 30250). To provide evidence favouring heterogeneity by sex, we additionally included an interaction term between the protein level and sex in the analyses on incident CAD, adjusted for the confounders.^23^ Unless otherwise stated, we corrected for multiple testing using the false discovery rate (FDR; Benjamini-Hochberg).

For the analysis of enriched biological pathways in our data, we used the integrated biological database Metascape (http://metascape.org).^24^ In short, Metascape facilitates the translation of large gene sets to involvement in biological processes via computed overrepresentation. More specifically, Metascape incorporates multiple ontologies for enrichment analysis and eliminates redundancies following hierarchical clustering of terms based on enrichment. The platform leverages >40 independent knowledgebases, among which the ontology resources Molecular Signatures Database (MSigDB), Kyoto Encyclopedia of Genes and Genomes (KEGG), Gene Ontology (GO) knowledgebase, Reactome Pathway Database and WikiPathways.

#### 2.1.2 Mendelian randomization

To provide evidence favouring possible causal associations, we performed two-sample MR analyses. For the single-nucleotide polymorphisms (SNP)-exposure associations, we used the previously reported data from the SNPs associated with the protein levels from the UK Biobank.^10^ From these, we selected all independent lead SNPs associated with protein levels (both cis and trans SNPs) with a SNP-exposure *P*-value <1.7e−11, considering multiple testing for the number of proteins in the genome-wide association study. Only independent genetic instruments were considered for further analyses when the R^2^ was below 0.0001 in the European reference panel, limiting potential bias caused by overlapping genetic effects. As SNP-outcome datasets, we used summary-level genome-wide association data from the Coronary ARtery Disease Genome wide Replication and Meta-analysis plus the Coronary Artery Disease Genetics consortium (CARDIoGramplusC4D) (60 801 cases and 123 504 controls),^25^ UK Biobank (122 733 cases and 424 528 controls) and freeze 9 from the Finngen biobank (https://www.finngen.fi/en) (43 518 cases and 333 759 controls),^26^ resulting in a total of 227 052 cases and 881 791 controls. In each dataset, CAD was defined as previously described in this methods section (I20, I21, I22, I24, I25). With the exception of the CARDIoGRAMplusC4D dataset, which comprised 23% of non-European ancestry participants, all other datasets only used participants of European ancestry. The studies contributing data to these (meta-)analyses have been approved by the necessary local medical ethics committees, and all participants contributing to these efforts provided written informed consent. For the purpose of the present study, we only used the summary-level data and no individual-level data. The sex-specific analyses were performed using data from the FinnGen and UK Biobank cohorts. In FinnGen, the male cohort included 218 285 individuals, of whom 39 646 were CAD cases, while the female cohort included 282 064 individuals, with 17 004 CAD cases. In the UK Biobank, the male cohort comprised 22 323 CAD cases and 181 500 controls, whereas the female cohort included 10 124 CAD cases and 229 507 controls.

##### 2.1.2.1. Mendelian randomization meta-analysis

The primary analysis for MR employed inverse-variance weighted (IVW) regression analysis, assuming the absence of invalid genetic instruments such as directional pleiotropy.^27^ All MR analyses utilized the R-based package ‘TwoSampleMR’ (version 0.6.11). The mean effect estimate was derived separately from each outcome database through fixed-effect IVW meta-analysis of Wald ratios (gene-outcome [logodds] divided by gene-exposure associations) estimated for each instrumental variable.^28^ Results were expressed as odds ratios (ORs) for CAD risk. Under the assumption of met MR criteria, this odds ratio served as an estimate of the causal effect of the exposure on the outcome.

To assess potential violations of main MR assumptions (relevance, independence and exclusion restriction) stemming from directional pleiotropy, MR-Egger regression analyses and weighted-median estimator analyses were performed.^28–30^ The MR-Egger's intercept estimated the average pleiotropic effect across genetic variants, with a (significant) non-zero value indicating bias in the IVW estimate.^29^ The weighted-median estimator yielded a consistent valid estimate if at least half of the instrumental variables were valid.^30^ All analyses were performed separately for the individual GWAS summary datasets and subsequently meta-analysed using fixed-effects with the R-based ‘rmeta’ package (version 3.0).

#### 2.1.3 ELISAs and plasma samples MISSION!

Plasma-EDTA samples obtained from the MISSION! Study,^31^ were used for the quantification of specific targets in blood using ELISA. These samples were collected immediately upon hospital arrival, prior to the procedure, from male (*n* = 40) and female (*n* = 40) patients, as described previously.^31^ All patients presented with symptoms of acute myocardial infarction within <9 h before arrival at the catheterization laboratory and had an electrocardiogram-confirmed ST-elevation myocardial infarction (STEMI). To diminish variation, patients with diabetes were excluded from sample selection. ELISAs were performed according to the manufacturers’ protocol for the targets AKT2 (ab208986, Abcam, UK), SKAP2 (abx383214, Abbexa, UK), SPON1 (STJE0012317, St. John's Laboratories, UK), LDLR (EHLDLR, Thermo Fisher Scientific, USA) and CTSH (EH75RB, Thermo Fisher Scientific, USA).

#### 2.1.4 Athero-express biobank

##### 2.1.4.1. Study population and data collection

All patients included in this analysis are part of the Athero Express biobank, which includes individuals from the University Medical Center Utrecht and St. Antonius Hospital, Nieuwegein, who have undergone carotid endarterectomy.^32^ All participants provided informed consent, and the study complies with the ethical principles of the Helsinki Declaration, with approval from the ethics committees of both hospitals.^33^

The study cohort was selected based on the availability of plasma samples, multi-omics data and a history of carotid endarterectomy, while ensuring a balanced representation of male and female participants. Patients with missing data on clinical risk factors or the study endpoint, MACE, were excluded. MACE outcomes were assessed over a three-year longitudinal follow-up period.

##### 2.1.4.2. Patient characteristics

Patient characteristics were assessed to provide an overview of the study population and to account for potential confounding factors. The baseline characteristics included age, sex, body mass index (BMI), glomerular filtration rate (GFR), diabetes status and smoking status. These variables were selected based on their known association with cardiovascular outcomes and their relevance to the study.

##### 2.1.4.3. Olink data

Plasma protein levels were measured in 426 participants using Olink Explore panels, covering Cardiometabolic I & II, Immunology I & II, Neurology I & II and Oncology I & II. This approach enabled the measurement of 2938 proteins in total. From this dataset, the following top 10 sex-specific candidate targets were selected for further investigation in the Athero Express (AE) cohort: CDKN2D, CTF1, CTSH, FADD, MAP2K6, MYH9, SKAP2, AKT2, LDLR, SPON1.

After handling missing clinical data, 384 participants were included in the final analysis. The cohort had a mean age of 69.82 (9.14) years, a mean BMI of 26.36 (4.1) and a mean GFR of 71.62 (19.64). At baseline, 75 participants (19.53%) had diabetes, and 145 (37.76%) were smokers. Written informed consent was obtained from all participants.

##### 2.1.4.4. Cox regression analysis

We performed two types of Cox proportional hazards regression analyses to assess the association between protein levels and time to MACE. First, univariate Cox regression models were fitted for each protein, including an interaction term with sex (protein * sex), to examine sex-specific associations without adjustment for clinical risk factors.

Subsequently, multivariate Cox regression models were performed, adjusting for established cardiovascular risk factors: age, body mass index (BMI), diabetes status, glomerular filtration rate (GFR) and smoking status. Specifically, these models retained the protein-by-sex interaction term to evaluate whether the sex-specific associations remained after controlling for potential confounders, and no other interaction terms were included.

For both analyses, hazard ratios (HRs) and 95% confidence intervals were calculated. The protein-by-sex interaction term was used to quantify and test for sex-specific differences in the association between protein levels and MACE risk.

##### 2.1.4.5. ROC curve analysis

To assess the predictive value of each protein for MACE events, we performed time-dependent Receiver Operating Characteristic (ROC) curve analyses stratified by sex. Using the timeROC package (version 0.4), we calculated ROC curves at 2.99 years of follow-up. For each protein, the direction of prediction was determined by the sex-specific coefficients obtained from the Cox regression models, ensuring proper handling of positive and negative associations.

Area Under the Curve (AUC) values were calculated separately for males and females. To test for significant sex differences in predictive performance, we conducted permutation tests (1000 permutations) by randomly reassigning sex labels while maintaining the original male-to-female ratio. The empirical *P*-value was calculated as the proportion of permutations where the absolute difference in AUC between sexes exceeded the observed difference. Confidence intervals for the ROC curves were estimated using confint (stats package, 3.6.2).

All statistical analyses were performed in R (version 4.4.0). Cox regression analyses were conducted using the ‘survival’ package (version 3.7.0), with the coxph() function implementing the proportional hazards regression model. Visualization of results was performed using the 'forestplot' package (version 3.0.6) to create forest plots of hazard ratios and their confidence intervals. Visualization of the ROC curves was performed with custom plotting functions using base R graphics.

#### 2.1.5 Druggability analyses

To evaluate target druggability, we used the machine learning platform DrugnomeAI to calculate aggregated probability scores based on a combined clinically approved (Tclin) and clinical-phase (Tier 1) drug candidate model.^34^ DrugnomeAI is an adaptation of mantis-ml, a machine learning framework, based on a stochastic semi-supervised learning approach.

Essentially, DrugnomeAI employs a process in which the machine learning algorithm learns from both labelled and unlabelled data in a probabilistic manner. Multiple resources have been integrated in the assessment of target druggability within DrugnomeAI, among which databases on protein-protein, drug-gene and chemical-gene interactions such as Pharos, Drug Gene Interaction Database (DGIdb) and Comparative Toxicogenomics Database (CTDbase).

### 2.2 Data availability

The present study has been conducted using the UK Biobank Resource (Application Number 56340) that is available to researchers. Summary data are available from the original sources. Summary-level data used for the two-sample Mendelian Randomization analyses are available online through the infrastructure of the R package twosampleMR or on the website of the FinnGen Biobank (https://www.finngen.fi/en).

## 3. Results

### 3.1 Primary analysis UK Biobank

#### 3.1.1 Summary information

From the UKB-PPP project cohort, a total of 40 829 individuals were included free-of-CAD at baseline and in the first year after inclusion, with a mean age of 56.9 (standard deviation 8.10) years, 55.3% women and a mean BMI of 27.0 kg/m^2^ (*Table 1*). Anti-hypertensive, cholesterol-lowering and insulin medication use at recruitment was 19.1%, 14.6% and 0.9% respectively, together with 7.3% of total participants being diagnosed with non-insulin-dependent diabetes mellitus. Moreover, there was a mean TDI of −1.52 (3.01) points and 44.6% of the individuals were past or current smoker (*Table 1*).

**Table 1:** Baseline characteristics of the study population

	Baseline (*n* = 40 829)	*Women* *(n* = *22 590)*	*Men* *(n* = *18 239)*
**Sex**			
Female (%)	22 590 (55.3%)		
Male (%)	18 239 (44.7%)		
**Age at recruitment (years)**			
Mean (SD)	56.9 (8.10)	56.8 (8.01)	57.0 (8.22)
**Body mass index**			
Mean (SD)	27.3 (4.71)	27.0 (5.08)	27.7 (4.16)
**Anti-hypertensive medication at recruitment**			
Yes (%)	7809 (19.1%)	3803 (16.8%)	4006 (22.0%)
**Cholesterol-lowering medication at recruitment**			
Yes (%)	5975 (14.6%)	2512 (11.1%)	3463 (19.0%)
**Insulin use at recruitment**			
Yes (%)	374 (0.9%)	166 (0.7%)	208 (1.1%)
**Non-insulin-dependent diabetes mellitus**			
Yes (%)	2996 (7.3%)	1249 (5.5%)	1747 (9.6%)
**Townsend deprivation index (socio-economic)**			
Mean (SD)	−1.52 (3.01)	−1.54 (2.95)	−1.56 (2.90)
**Smoking**			
Past and current (%)	18 202 (44.6%)	9101 (40.3%)	9101 (49.9%)

SD, standard deviation.

#### 3.1.2 Proteomic associations with CAD

After a median follow-up time of 13.7 (IQR: 13.1, 14.4) years, there were 3155 incident CAD cases, of which 38.7% were women. In sex-combined multivariable-adjusted analyses, 1060 out of 2922 proteins were associated with incident CAD after correction for multiple comparisons (FDR) (*Figure 1A*, Supplementary material online, *Figures S1* and *S2*, Supplementary material online, *Table S2*). Separate sex-stratified analyses identified 440 female- and 32 male-specific CAD proteins (*Figure 1A, B, C*, Supplementary material online, *Tables S3* and *S4*). An additional 96 proteins were observed to be associated with incident CAD in the sex-combined analyses, not observed in the sex-stratified analyses due to their limited effect size. Adjustment nor restriction of women for post-menopausal status did substantively change the results (see Supplementary material online, *Figure S3*, Supplementary material online, *Table S5*). Similarly, adjustment for baseline blood cell counts, including basophil, eosinophil, lymphocyte, monocyte, neutrophil and reticulocyte counts, did not materially affect the proteomic associations (see Supplementary material online, *Figure S4A*).

**Figure 1:**
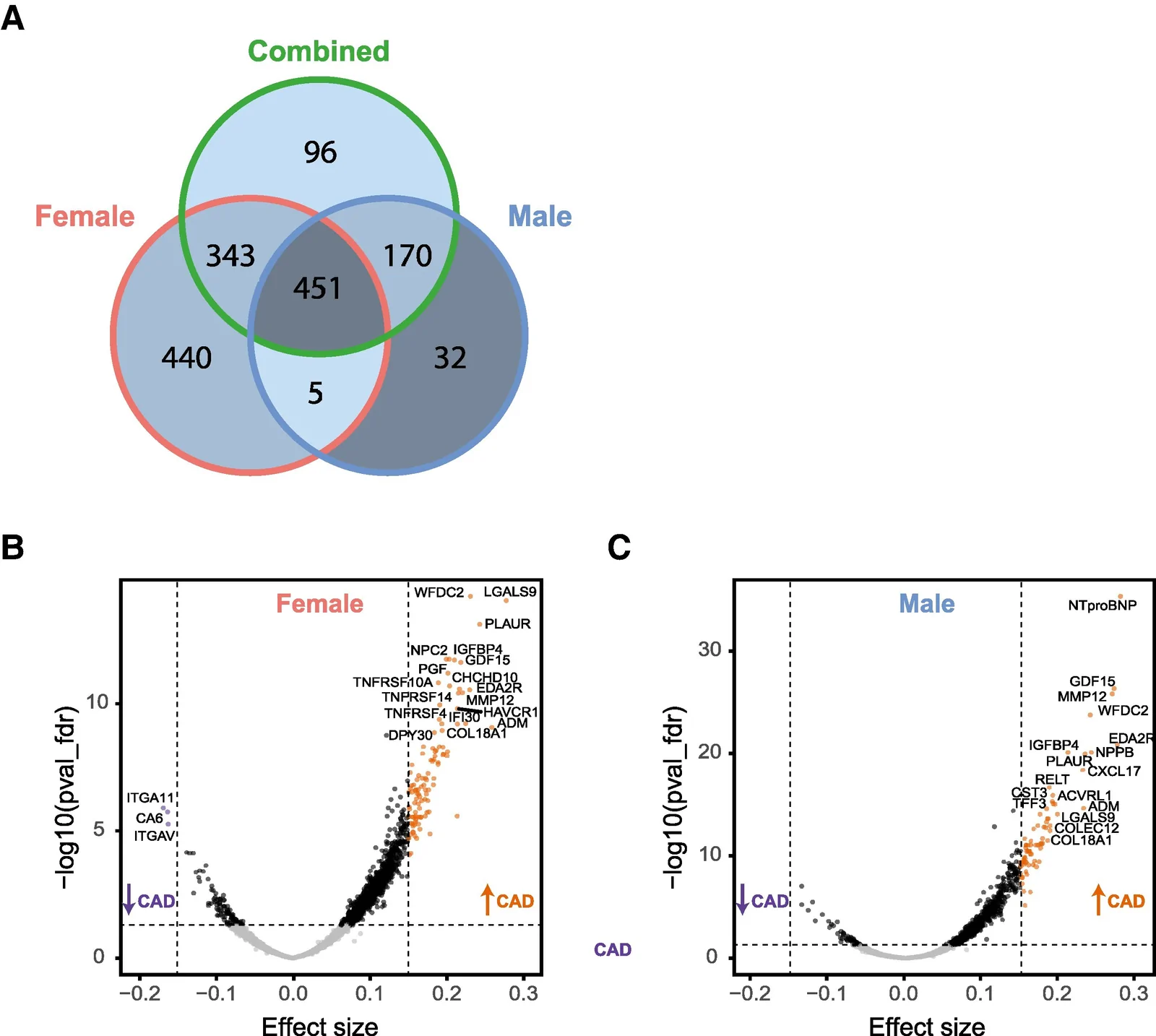
Proteomic associations for incident CAD. (*A*) Venn diagram of proteins associated with CAD for the sex-combined, female-specific and male-specific Cox-proportional hazard models. (*B*, *C*) Volcano plot representing the effect size of female- and male-specific plasma-proteomic profiles for CAD (FDR < 0.05, abs(β) ≥ 0.15). Orange dots denote positive significant associations with incident CAD. The proteins were part of the Cardiometabolic, Cardiometabolic_II, Inflammation, Inflammation_II, Neurology, Neurology_II, Oncology and Oncology_II panels of the Olink Explore 3072 platform. FDR: false discovery rate. Number of included individuals in the Cox-proportional hazard models: 40 829. Models were fitted in R version (3.6.1) using the survival package and adjusted for considered confounders.

For graphical reasons only, we filtered for abs(β) ≥ 0.15 across the combined, female- and male-specific analyses, resulting in 131 proteins of interest. Considering this cut-off, this resulted in 71 combined, 43 female-specific and 17 male-specific hits for heatmap visualization of the sex-specific plasma-proteomic signatures with the largest effect sizes (*Figure 2*).

**Figure 2:**
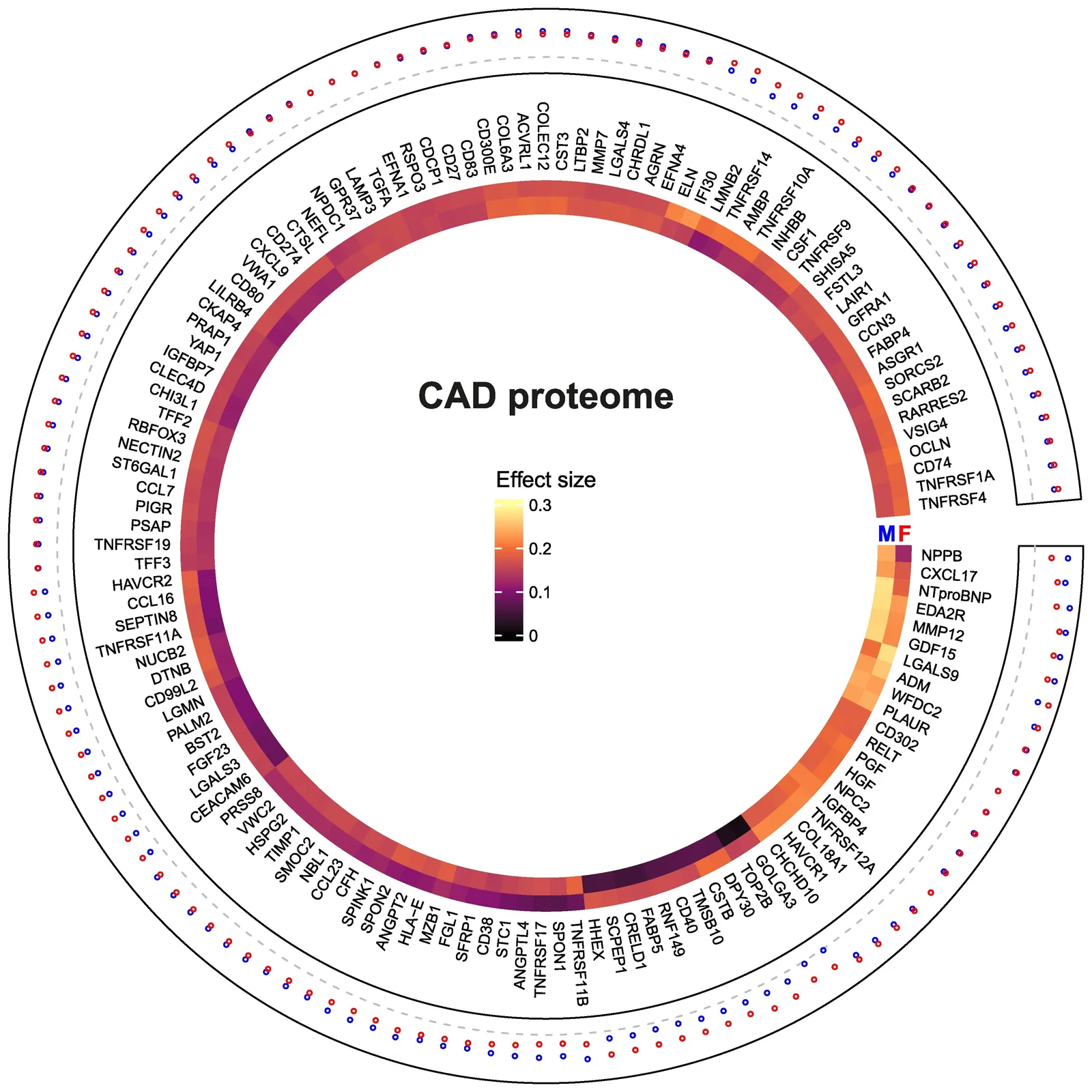
Circular heatmap for proteins associated with increased hazard for incident CAD for men and women. Average effect sizes per protein are represented by the heatmap layer women (outer) and men (inner) respectively, based on the female-specific and male-specific Cox-proportional hazard models in UK Biobank General clustering was plotted in a counter-clockwise fashion around the heatmap, based on increasing effect sizes and computed sex differences. Number of included individuals in the Cox-proportional hazard models: 40 829.

#### 3.1.3 Sex-specific proteomic pathways

Based on the complete CAD-incidence-associated plasma proteome in women and men (*Figure 1B* and *C*), we performed biological pathway analyses. This revealed sex-specific differences and similarities in proteomic pathways associated with incident CAD (*Figure 3*, Supplementary material online, *Figure S1B*, Supplementary material online, *Tables S6* and *S7*). Notable distinctions were observed between both sexes, including angiogenic (tube morphogenesis) and hormonal response (insulin-like growth factor response) components in men, and the innate immune response and programmed cell death regulation in women (*Figure 3A, B*). Moreover, we also found multiple shared pathways between both sexes, among which those corresponding to the matrisome, cytokine-cytokine receptor interactions, regulation of cell migration, neutrophil degranulation and enzyme-linked receptor protein signalling.

**Figure 3:**
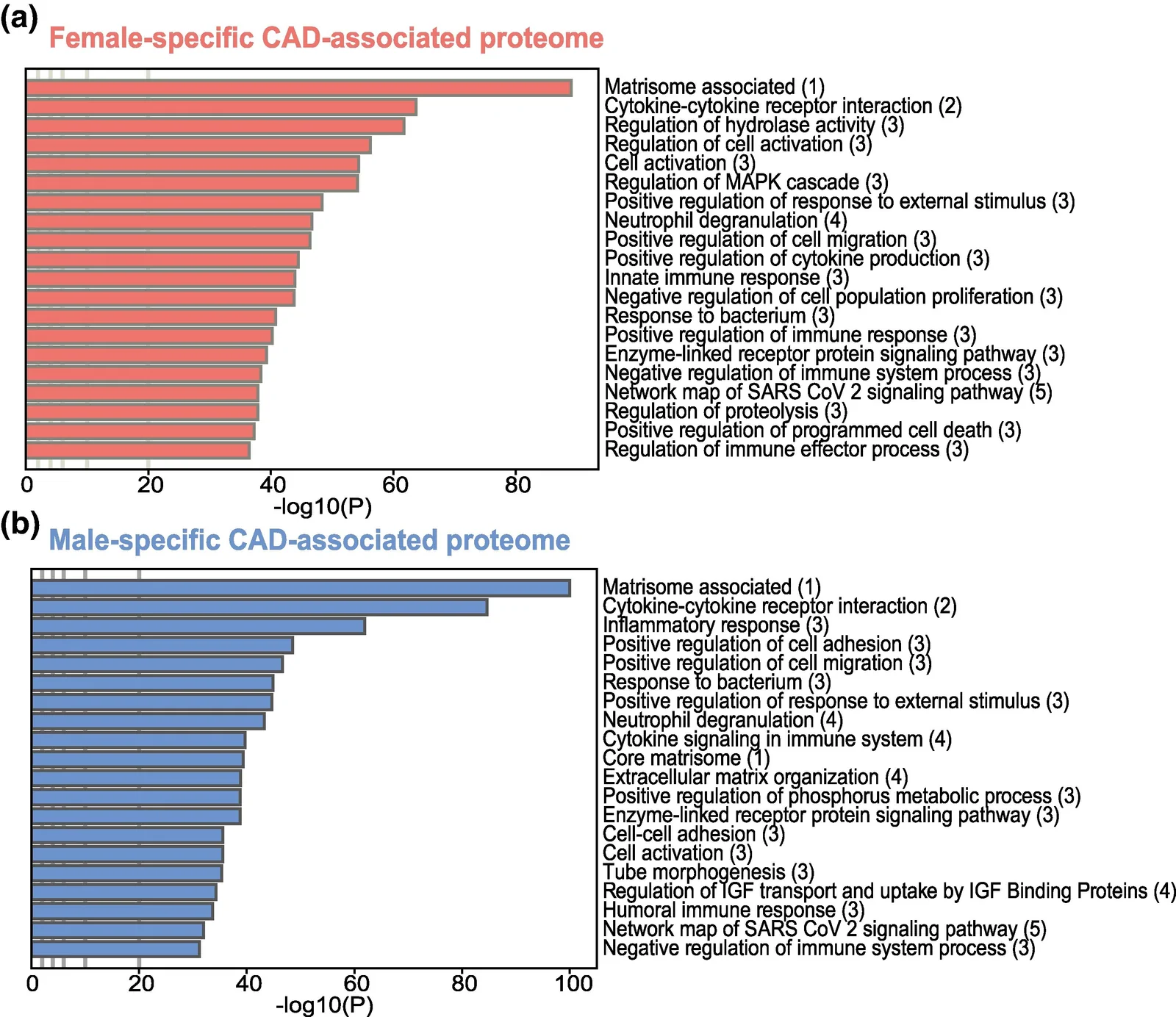
Sex-specific proteome analyses for CAD per pathway analyses for CAD incidence. (*A*, *B*) Pathway analyses of respectively the female and male CAD-associated proteome. Multiple ontology sources were included to cluster all enriched terms into groups via the integrated biological database Metascape. Number of included individuals in the Cox-proportional hazard models: 40 829. *(1) MSigDB: Molecular Signatures Database; (2) KEGG: Kyoto Encyclopedia of Genes and Genomes; (3) GO: Gene Ontology knowledgebase;(4) Reactome; and (5)WP: WikiPathways*.

#### 3.1.4 Sex-protein interaction analyses

Based on direct sex-protein interaction analyses (male vs. female),^23^ we found 703 proteins to be different between men and women (FDR < 0.05; *Figure 4A*, Supplementary material online, *Tables S8* and *S9*, Supplementary material online, *Figure S4B*). On the basis of the proteins with different effect sizes between men and women, we performed pathway analyses and subsequent network auto-clustering and identified 15 independent pathway clusters (*Figure 4B*, Supplementary material online, *Table S10*). In general, the 15 clusters represented differences between the CAD-associated proteomic signatures of men and women pertaining to innate and adaptive immunity, matrix remodelling, growth factor responses, apoptosis modulation and lipid management. The three largest clusters were ‘response innate immune’, ‘pathogenic infection virus’ and ‘cysteine endopeptidase regulation’ (*Figure 4B*).

**Figure 4:**
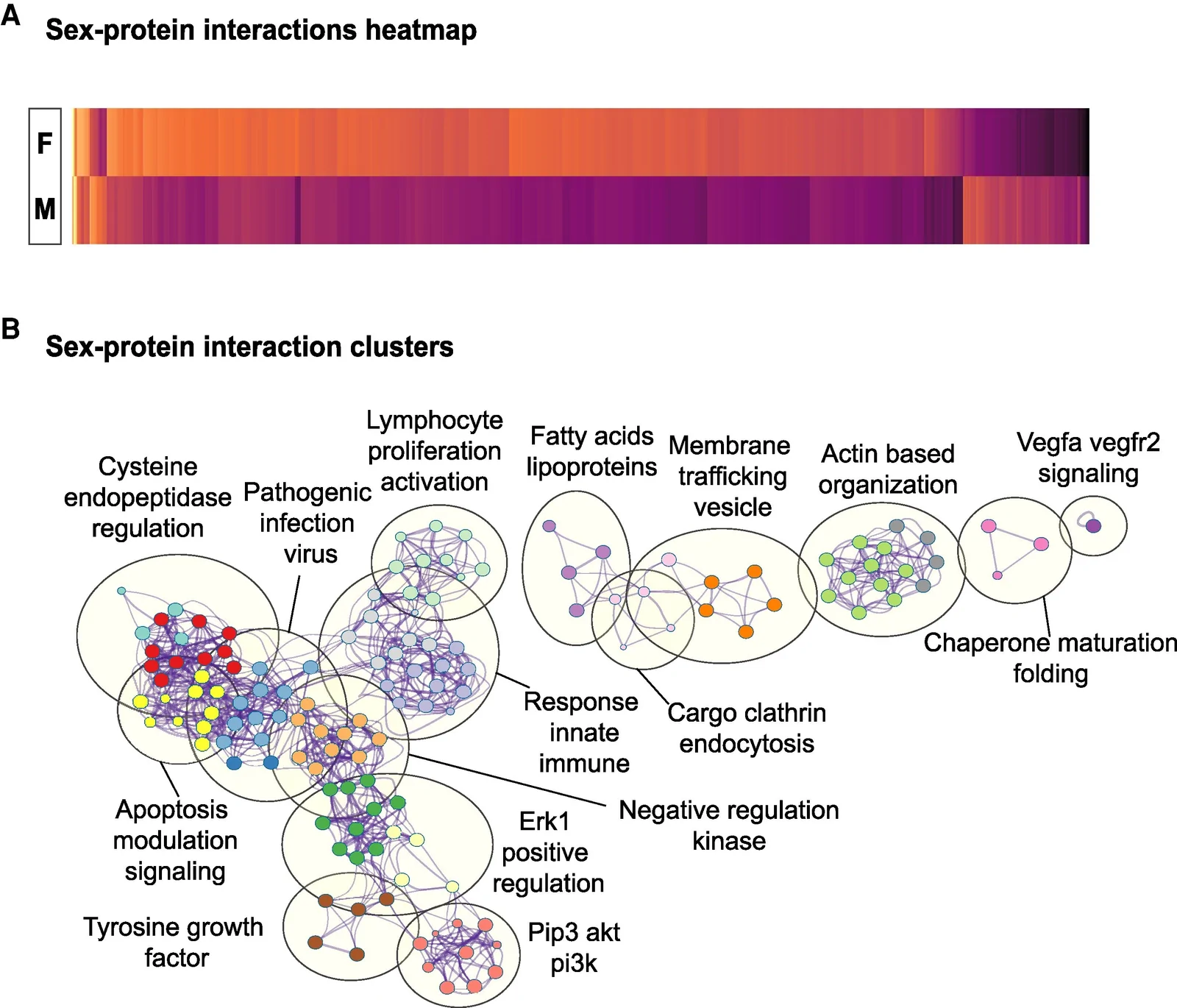
Sex-protein interactions. (*A*) Sex-protein interaction heatmap of significantly different proteins associated with CAD onset between men and women (*n* = 703 proteins), based on an additional interaction term between protein level and sex in multivariable adjusted Cox model for incident CAD (FDR<0.05). (*B*) Network auto-clustering based on significant interaction clusters for incident CAD. Nodes represent individual pathways corresponding to the higher order cluster. FDR: false discovery rate. Analyses were performed in R version 3.6.1 Number of included individuals in the Cox-proportional hazard models: 40 829.

### 3.2 MR meta-analysis

We performed two-sample MR analyses on 2346 out of the 2922 proteins for which genetic instruments have been previously reported and were suitable for MR analyses in 1 108 843 individuals.^20^ From the MR analyses, we observed evidence for possible causal associations for 219 proteins with increased CAD risk in the meta-analysis of the three individual CAD GWAS (*Figure 5A*, Supplementary material online, *Table S11*).

**Figure 5:**
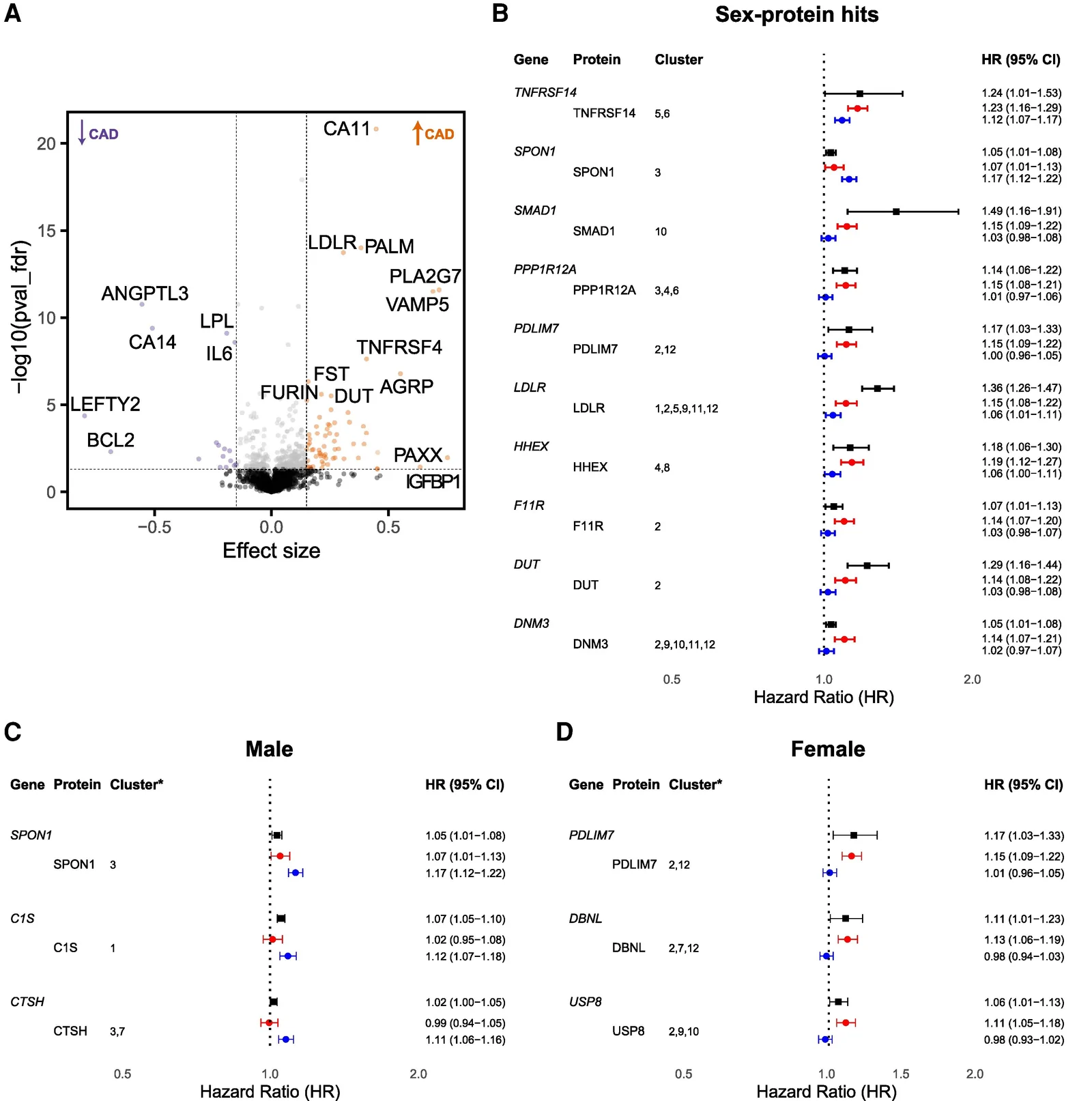
Causal evidence for sex-specific target associations with CAD. (*A*) Volcano plot of genetic MR associations with CAD in the combined UK Biobank-FinnGen-CARDIoGRAMplusC4D cohort. Positive and negative associations are shown (FDR<0.05, abs(β) ≥ 0.15). The primary Mendelian randomization analysis used inverse variance weighted regression, implemented in R using the TwoSampleMR package (0.6.11), and results from the individual datasets were combined using fixed effects meta-analysis with the rmeta package (3.0). (*B*) Forest plot depicting the top 10 target protein hazard ratios resulting from the sex-protein interaction analyses, as causally validated by the MR meta-analysis data (black) and jointly sorted for women and men. (*C*, *D*) Forest plot depicting the top three protein hazard ratios for CAD, as sorted for male- (*C*) and female-specific (*D*) differential top hits. Number of included individuals in the combined MR analyses: 1 108 843. Number of included individuals in the Cox-proportional hazard models: 40 829. *Clusters according to the interaction-analyses: *1: response innate immune, 2: actin based organization, 3: cysteine endopeptidase regulation, 4: negative regulation kinase, 5: pathogenic infection virus, 6: erk1 positive regulation, 7: apoptosis modulation signalling, 8: lymphocyte proliferation activation, 9: membrane trafficking vesicle, 10: tyrosine growth factor, 11: cargo clathrin endocytosis, 12: fatty acids lipoprotein, 13: chaperone maturation folding, 14: vegfa vegfr2 signalling*.

First, from the 219 proteins with evidence for potential causality, we identified the top 20 sex-combined hits associated with CAD across for the mutual average of female and male hazard ratios as shown in (see Supplementary material online, *Table S12*). Next, to identify the sex-specific proteins with genetic evidence for causality for CAD, we examined the 219 MR hits for occurrence within the 703 proteins from the sex-protein interaction analyses (see Supplementary material online, *Table S13*, *Figure 4A, B*). In total, we identified 59 out of the 703 hits that pertained to varying groups of sex-protein interaction clusters, of which the ten with the highest combined hazard ratio are shown in *Figure 5B* (see Supplementary material online, *Table S13*).

When considering the highest absolute differences between both sexes, Spondin 1 (SPON1), Complement C1s (C1S) and Cathepsin H (CTSH) emerged as top hits for men, while PDZ And LIM Domain 7 (PDLIM7), Drebrin-like (DBNL) and Ubiquitin-specific peptidase (USP8) were prominent within the specific top hits for women. Further examination revealed sex-specific nuances of the data within the framework of the 15 pathway clusters (*Figure 4B*, Supplementary material online, *Table S13*).

### 3.3 Sex-stratified MR

We subsequently performed sex-stratified MR in 943 803 individuals on the 59 proteins with evidence for causality in the sex-combined MR analyses and evidence for sex differences in the regression analyses, followed by direct female-to-male interaction analyses.^23^ From these analyses, we identified seven proteins with at least suggestive evidence for sex- specific causality in CAD, appreciating somewhat limited statistical power, especially in the female analyses (*P* < 0.1; *Table 2*, Supplementary material online, *Table S14*). More specifically, we found CTSH in men and CDKN2D, MYH9 and SKAP2 in women to be relevant from our sex-stratified MR. Interestingly, while CTF1, FADD and MAP2K6 exhibited significant plasma proteomic associations with CAD specific to women, our genetic evidence pointed towards a male-specific effect for these proteins (*Table 2*).

**Table 2:** Clinical setting data and druggability of top sex-specific hits

Target	Sex-protein direction	Sex-specific MR		Plasma concentration AMI in MISSION! (ELISA, ng/mL)		Cox regression analysis for secondary MACE in AE Biobank		Druggability score
		Women HR (95% CI)	Men HR (95% CI)	Women Median (IQR)	Men Median (IQR)	Women HR (95% CI)	Men HR (95% CI)	
**AKT2**	Female	1.30 (1.08–1.58)	1.07 (0.90–1.27)	1.41 (0.62–1.98)	1.50 (0.95–2.47)	1.12 (0.65–1.91)	1.03 (0.73–1.45)	0.9936
**CTSH**	Male	0.98 (0.95–1.01)	1.02 (0.99–1.04)	1.015 (0.75–2.25)	0.95 (0.66–4.11)	0.91 (0.57–1.46)	1.12 (0.71–1.77)	0.5118
**LDLR**	Female	1.04 (0.94–1.16)	1.08 (0.93–1.24)	18.73 (13.69–24.35)	15.68 (11.09–22.37)	1.34 (0.61–2.95)	0.99 (0.53–1.83)	0.8542
**SKAP2**	Female	1.37 (1.14–1.64)	1.07 (0.91–1.25)	4.52 (3.53–4.52)	5.38 (4.56–5.90)	1.14 (0.83–1.58)	1.01 (0.81–1.26)	0.047
**SPON1**	Male	0.99 (0.93–1.06)	1.04 (1.00–1.08)	Undetectable	Undetectable	2.12 (1.29–3.50)	1.40 (0.94–2.09)	0.09470
**CDKN2D**	Female	1.21 (1.07–1.37)	1.04 (0.94–1.16)	n.a.		1.04 (0.71–1.53)	1.00 (0.79–1.27)	0.2891
**CTF1**	Female	0.96 (0.77–1.19)	1.22 (1.05–1.42)	n.a.		0.97 (0.56–1.68)	1.20 (0.84–1.71)	0.6142
**FADD**	Female	0.91 (0.76–1.10)	1.11 (0.99–1.24)	n.a.		1.18 (0.69–2.01)	1.01 (0.72–1.45)	0.4895
**MAP2K6**	Female	0.87 (0.68–1.11)	1.16 (0.98–1.37)	n.a.		1.21 (0.77–1.88)	0.96 (0.70–1.31	0.978
**MYH9**	Female	1.27 (1.11–1.45)	1.01 (0.9–1.14)	n.a.		1.14 (0.77–1.69)	1.07 (0.81–1,40)	0.1045

IQR, inter-quartile range; CI, confidence interval; MR, mendelian randomization; HR, hazard ratio; MACE, major adverse cardiovascular events.

### 3.4 Sex-specific hits in MISSION! and athero-express cohorts

To further study the biological application of the identified targets, we investigated their association in the context of acute myocardial infarction and major adverse cardiovascular events. In detail, we selected ten of the most distinct sex-specific proteins based on sex-protein interactions and combined or sex-specific MR analyses (*Table 2*).

Proteomic expression data from the UK Biobank indicated that none of our selected targets exhibited relevant baseline differences in expression levels between men and women in the total general population (*n* = 40 829; Supplementary material online, *Table S14*). However, in our MISSION! cohort of patients with acute myocardial infarction (AMI), notable sex differences were observed in the median and/or distribution of plasma concentrations of AKT2, CTSH, LDLR and SKAP2 (*Table 2*, *Figure 6A–D*, Supplementary material online, *Table S15*). For SPON1, 95% of samples had immeasurably low target levels (*Table 2*).

**Figure 6:**
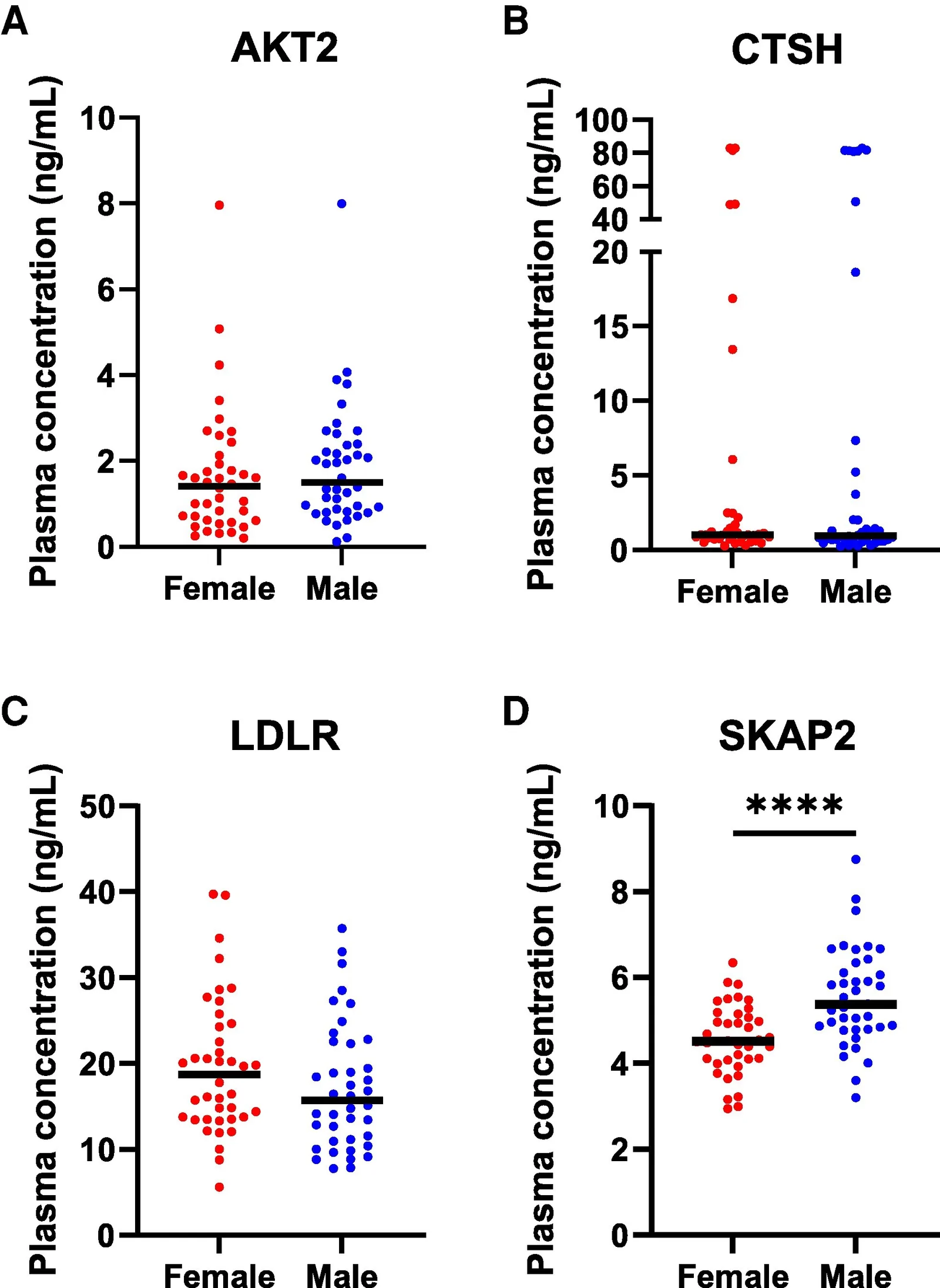
Plasma concentration of top sex-specific hits in the plasma of women (*n* = 40) and men (*n* = 40) with acute STEMI from the MISSION! study. ELISA results are shown for AKT2 (*A*), CTSH (*B*), LDLR (*C*) and SKAP2 (*D*), with between sex differences assessed using unpaired two-sided statistical testing. Plasma EDTA samples were obtained immediately upon hospital arrival, before coronary intervention, and ELISAs were performed according to the manufacturers’ protocols. Plasma concentrations of the fifth target, SPON1, were undetectably low for 95% of samples. Number of included individuals in statistical testing: 80.

We next analysed a cohort of 384 patients (173 female, 211 male) from the Athero-Express biobank who underwent carotid endarterectomy and were followed for up to three years, with major adverse cardiovascular events (MACE; *n* = 45; 17 female, 28 male) as a secondary outcome (see Supplementary material online, *Table S16*). Whereas no proteins were identified with a significant protein-sex interaction effect in Cox regression analyses, sex-stratified analyses revealed that SPON1 had a higher hazard ratio (HR) in female patients (*Table 2*). This finding remained robust after adjustment for key risk factors (see Supplementary material online, *Figure S5*; Supplementary material online, *Tables S17*–S20).

### 3.5 Top target druggability

First, we assessed druggability of the 59 proteins with both evidence for sex differences in the regression analyses and for causality in the sex-combined MR (see Supplementary material online, *Table S21*). Machine learning-derived aggregated druggability probability scores were based on a combined clinically approved (Tclin) and clinical-phase (Tier 1) drug candidate model. From this integrated model, 14 out of 59 proteins were identified with high druggability scores surpassing the threshold of 0.5. Further analyses were performed on the previously identified subset of ten of the most distinct sex-specific proteins based on sex-protein interactions and combined or sex-specific MR (*Table 2*). In this subset, CTSH demonstrated druggability with a 0.51 score for men (*Table 2*). In the context of the female top hits, limited druggability was found, with the first druggable target being CDKN2D (0.2891) (*Table 2*, Supplementary material online, *Table S21*).

Next, we assessed druggability of our top 20 sex-combined targets, which showed evidence for causality in men as well as women (see Supplementary material online, *Table S12*). Six combined targets emerged with high druggability probability scores above the threshold of 0.5. Notably, TNFRSF4, CSF1, FURIN, CD74, TNFSF13 and MSR1 demonstrated high potential for therapeutic intervention for men and women together (see Supplementary material online, *Table S12*).

## 4. Discussion

Through integrated genomic and proteomic approaches, we observed the presence of sex-specific differences in the association of the plasma proteome with incident CAD. Differences between men and women were observed in pathways related to matrix organization, cytokine interactions, regulation of innate and adaptive immune cells, angiogenesis and growth factor signalling related to incident CAD. Furthermore, we provided evidence favouring causal associations in sex-combined and sex-specific study samples using MR approaches. These findings provide insights into sex-specific pathophysiological mechanisms and implications for incident CAD and allow for the identification of potential druggable targets unique to men and women.

Despite apparent differences in top target findings of previous plasma proteomic approaches,^10,12,35,36^ we found potential evidence for the contribution of shared biological pathways leading to CAD. Notably, from our sex-protein interaction analyses it appeared that pathways, such as vascular endothelial growth factor (VEGF) signalling, may be associated with sex differences in CAD development. Previously, we and others have shown that dysfunctional VEGF signalling is involved in leaky plaque neovessels and can subsequently contribute to lesion destabilization.^37,38^ Non-mature neovessels promote plaque development as the endothelium functions as a significant entrance route for immune cells via NF-κB promotion and subsequent TNF signalling. Endothelial dysfunction has been described in both sexes within the context of atherosclerotic disease. Men are more likely to suffer from intraplaque haemorrhage secondary to neovessel-rupture, whereas women more often present with coronary microvascular dysfunction (CMD) and subsequent angina with nonobstructive coronary arteries.^39,40^ This is noteworthy, considering that female hormone signalling has been associated with CMD,^41^ while oestrogen replacement therapy has not been found effective to halt classical obstructive atherosclerotic plaque progression in post-menopausal women with ischaemic heart disease.^42,43^

After integration of large-scale plasma proteomics with MR analyses, we identified 59 sex-specific associations with evidence supporting possible causality. Among these, congruent sex-specific MR evidence was observed for CTSH (cysteine protease) in men, and CDKN2D (cyclin-dependent kinase inhibitor), MYH9 (class II myosin) and SKAP2 (Src family kinase) in women. Further evidence from patients with AMI supported the observation of sex differences in the distribution of the protein plasma concentration of SKAP2, which has been suggested to regulate the inflammatory macrophage response in atherosclerosis.^44^ Although we found more proteins to be associated with incident CAD in women compared to men, despite the lower number of cases in women, the strongest female-specific associations lacked strong druggability evidence in current databases. The top hit in men, CTSH, was identified as a druggable target, and it is known that cysteine protease cathepsins may mechanistically affect atherogenesis and other vascular disease via their collagenolytic and elastolytic activity.^45^ Moreover, it has been described that CTSH may contribute to the early atherogenic function of LDL in the plaque.^46^

In addition to the sex-specific findings, our results also highlight shared risk factors, including the well-described target PCSK9, which may further substantiate the concept that men and women exhibit overlapping conventional (protein) risk factors. Considering that recent evidence suggests that sex-differential genetic regulation of circulating protein levels is uncommon,^16^ our observed sex-specific protein-CAD associations may reflect sex-dependent modulation of proteins in shared biological pathways, although mechanistic insight into these processes is currently limited and warrants further study.

For 6 out of the 20 of our strongest sex-combined CAD-associated hits (TNFRSF4, TNFSF13, CSF1, FURIN, CD74 and MSR1), we found evidence for druggability. Tumour necrosis factor levels have been previously associated with a higher risk of CAD, and specific drugs targeting TNFRSF4 and TNFSF13 have indeed been employed in clinical trials in patients with autoimmune diseases.^47–50^ FURIN, a member of the proprotein convertases, was recently found by Mazidi et al. as their most strongly associated protein with ischaemic heart disease^12^ and was previously linked to atherogenesis via upregulation of TNFSF13.^51,52^ CSF1 and MSR1 are known drivers of atherosclerosis through their roles in monocyte/macrophage recruitment, activation and foam cell formation.^53^ CD74, involved in antigen presentation, is targeted in haematologic malignancies (clinicaltrials.gov: nct02701985) and its degradation is linked to cathepsin S, a protease affecting atherosclerotic plaque stability.

Altogether, our findings point towards potential sex-specific pathways and factors in CAD development and suggest new drug candidates with evidence for causality among our identified hits. Importantly, elucidation of the precise biological pathways linking these proteins to sex-specific disease development requires targeted functional studies.

### 4.1 Strengths and limitations

The primary strength of our study is the large sample size and in particular the proportion of included women, enabling the analysis of a comprehensive pool of plasma proteins in association with CAD while correcting for multiple testing and confounders. The integration of genomics and proteomics provided evidence for sex-specific causality in large samples. The consistency across the different analytical frameworks used in the present study suggests the results are more likely observed due to biological differences between men and women in developing CAD rather than random noise and false-positive findings.

Limitations include the focus on individuals of European ancestry, due to a lack of sufficient numbers in the different other ancestry groups, and therefore these results apply to only that part of the population. It is important to validate these findings for individuals of non-European ancestry in future studies of sufficient size. Furthermore, in the multivariable-regression analyses, we excluded participants who developed CAD within the first year of follow-up to limit the potential influence of reverse causation. Nevertheless, this approach does not limit the potential presence of sex-specific residual confounding, although adjustment nor restriction of women for postmenopausal status did materially differs the results, suggesting such confounding might be limited.

The antibodies in the current proteomics approach are selected to identify epitopes in the circulation derived from known proteins, regardless of the original cellular localization of these proteins. Numerous epitopes are detected by this approach from proteins that play acknowledged intracellular roles (i.e. AKT2 and SKAP2 -cytoplasmic kinases-, and CTSH -a lysosomal protease-) or are present on the cell surface (i.e. LDLR -the LDL receptor-). For many of these protein derived epitopes, the functional significance of their presence in the circulation is unknown. For some intracellular proteins, the presence of epitopes in the circulation may be due to cellular leakage of complete proteins or degraded fragments. However, some cell surface and intracellular proteins may also undergo cleavage and/or modification and subsequent shedding or excretion. For example, for the LDLR, an enzyme responsible for shedding has been identified and the subsequent presence of the soluble ectodomain (sLDLR) was shown to inhibit plasma cholesterol levels and accelerate atherosclerosis in mice.^54^ However, for the majority of proteins the presence of their epitopes in the circulation is poorly understood and requires additional research.

Translational perspective Coronary artery disease (CAD) remains the leading cause of death in both men and women, yet sex-specific investigations of its onset remain limited despite differences in clinical presentation. In >40 000 participants, analysis of 2922 plasma proteins identified 440 female- and 32 male-specific associations with CAD, implicating distinct pathways such as innate immunity in women and angiogenesis in men. Using a triangulation approach combining sex-stratified MR, druggability prediction and external cohorts, we confirmed sex-specific targets including CTSH in men and TNFRSF4 in both sexes. These findings support sex-informed approaches to CAD and may help guide future tailored interventions.

## 5. Conclusion

In summary, by linking the sex-specific plasma proteome to CAD incidence and providing evidence for causal relations, we were able to identify female- and male-specific proteins along with their biological pathways. While a number of results showed discordant evidence between the multivariable-adjusted and Mendelian Randomization regression analyses between men and women, additional follow-up studies in well-characterized cohorts are required to provide further validation. These findings offer potential directions for developing targeted preventive and interventional strategies against CAD.

## Supplementary Material

cvag186_Supplementary_Data

## Data Availability

The present study has been conducted using the UK Biobank Resource (Application Number 56340) that is available to researchers. Summary data are available from the original sources. Summary-level data used for the two-sample Mendelian Randomization analyses are available online through the infrastructure of the R package twosampleMR or on the website of the FinnGen Biobank (https://www.finngen.fi/en).
